# Erythrocyte Vulnerability to Airborne Nanopollutants

**DOI:** 10.3390/toxics12010092

**Published:** 2024-01-21

**Authors:** Cristina Hermosillo-Abundis, Aracely Angulo-Molina, Miguel A. Méndez-Rojas

**Affiliations:** 1 Department of Chemical & Biological Sciences, Universidad de las Américas Puebla, San Andres Cholula, Puebla 72810, Mexico; anac.hermosilloas@udlap.mx; 2 Department of Chemical Biological Sciences, Universidad de Sonora, Hermosillo 83000, Mexico; aracely.angulo@unison.mx

**Keywords:** hemolytic activity, ultrafine particles, nanopollutants, erythrocyte membrane, soot, magnetite, silica

## Abstract

The toxicological impact of airborne polluting ultrafine particles (UFPs, also classified as nanoparticles with average sizes of less than 100 nm) is an emerging area of research pursuing a better understanding of the health hazards they pose to humans and other organisms. Hemolytic activity is a toxicity parameter that can be assessed quickly and easily to establish part of a nanoparticle’s behavior once it reaches our circulatory system. However, it is exceedingly difficult to determine to what extent each of the nanoparticles present in the air is responsible for the detrimental effects exhibited. At the same time, current hemolytic assessment methodologies pose a series of limitations for the interpretation of results. An alternative is to synthesize nanoparticles that model selected typical types of UFPs in air pollution and evaluate their individual contributions to adverse health effects under a clinical assay of osmotic fragility. Here, we discuss evidence pointing out that the absence of hemolysis is not always a synonym for safety; exposure to model nanopollutants, even at low concentrations, is enough to increase erythrocyte susceptibility and dysfunction. A modified osmotic fragility assay in combination with a morphological inspection of the nanopollutant–erythrocyte interaction allows a richer interpretation of the exposure outcomes. Membrane–nanoparticle interplay has a leading role in the vulnerability observed. Therefore, future research in this line of work should pay special attention to the evaluation of the mechanisms that cause membrane damage.

## 1. Introduction

The exposure to pollutants dispersed in the environment has been widely recognized as a potential health hazard. Different chemical substances have been linked to the increase in several health problems, such as cardiovascular disease, cancer, respiratory complications, diabetes, and even early neurodegeneration. Among them, particulate matter (PM) present in polluted air, usually classified according to its size range, is of current interest as its concentration and variety are increasing due to its anthropogenic activities. In particular, ultrafine particles (UFPs, also known as nanoparticles or nanopollutants), with sizes under 0.1 m (1–100 nm) are becoming a cause of concern due to their potential environmental and biological impacts as their small sizes facilitate their pass through different biological barriers, cell translocation, and improved biodistribution. Research has linked UFPs to several health issues, including cardiovascular pathologies, negative cognitive and psychiatric effects, immune-related diseases, neurodegenerative risk increases, and even developmental delays in children [1,2,3,4,5,6,7]. Due to their very small size, they can remain suspended for a longer time and can travel further distances, increasing the risk of exposure and the possible health effects derived from it [8]. Furthermore, unlike larger particles present in atmospheric pollution, ultrafine particles or nanopollutants are the only ones that can reach the systemic circulation [9], and their behavior inside the organism depends entirely on the physicochemical characteristics they exhibit (size, shape, charge, chirality, protein corona, etc.) [10,11,12]. Nanopollutants reach the atmosphere either as a byproduct of natural sources or anthropogenic activities; therefore, polluted air is a complex mixture involving a diverse variety of nanoparticles [13,14,15]. Moreover, the type and amount of pollutants present can vary depending on factors such as humidity, temperature, geographical location, and the strength of winds [5,16]. Considering such factors, some of the most common contaminants found in urban areas are silica nanoparticles, iron oxides, and carbon particles. Their presence is related to the urban high levels of construction, traffic, and industrial activity [15,17]. Airborne nanopollutants have shown higher toxicity than other sizes of particles, as they can induce oxidative stress, leading to inflammation and aggravating pre-existing respiratory or cardiovascular diseases [18]. PM and UFPs present in urban polluted air have been related to the development of neurodegenerative diseases such as Alzheimer’s disease (AD), Parkinson’s disease (PD), and brain disorders such as TAR DNA-binding protein (TDP-43) pathologies, as well as damage to the central nervous system when they move through the blood–brain barrier or damage blood vessels [10]. However, there are still many questions about the molecular mechanisms related to the biological effects and potential human impacts associated with environmental exposure to nanoparticles. For this reason, it is important to evaluate their toxicity and specific interactions with different tissues, organs, and systems.

**Figure 1:**
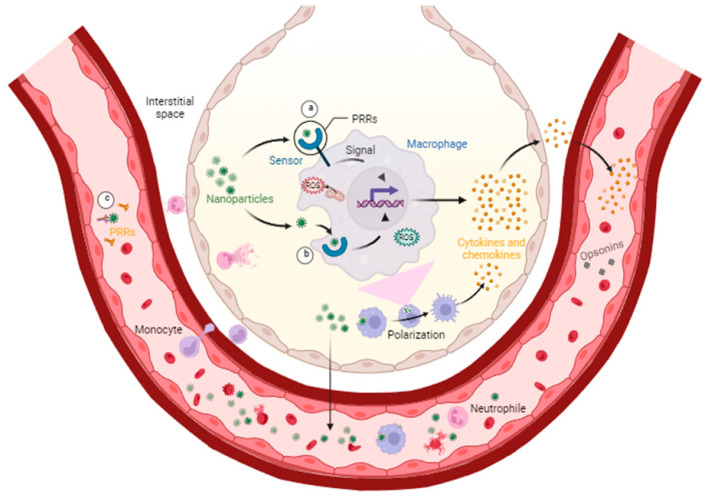
Alveoli–bloodstream interface. Macrophages in the alveoli have pattern recognition receptors (PRRs). These receptors are sensors that can be present on the surface of a cell (**a**), in intracellular spaces (**b**), and in the bloodstream (**c**). They can recognize arriving airborne nanoparticles inducing intracellular or extracellular signals that lead to changes in cytokines, chemokines, and reactive oxygen species (ROS) production. The signals can also induce changes in the phenotype of the macrophage (polarization) and other immune cells toward a proinflammatory type, and the soluble molecules produced can trigger the adaptive immune response through opsonins. Similar pathways can be activated by intracellular receptors when the macrophage phagocytes the nanoparticles. Once they reach the bloodstream, the nanopollutants can travel to every corner of the body.

Although humans can be exposed to nanopollutants through different routes like ingestion or deposition, inhaled nanopollutants can pass through the walls of the lungs and reach the bloodstream faster than other routes of exposure; once in the bloodstream, they can cause inflammation and damage to various organs and arteries in the body through the increased levels of reactive oxygen species, production of proinflammatory cytokines, complement activation, and adaptive immune system recruitment (see Figure 1) [19,20,21].

Although airborne nanoparticles’ health hazards for humans are clear, they are not being specifically assessed in the toxicology of air pollution, most of the data gathered come from nanomaterials carefully designed for biomedical applications where human safety becomes a critical concern [5,22,23,24]. The limited knowledge of potential nanopollutant interactions with erythrocytes is also centered on nanomaterials designed for biomedical applications. Engineered nanomaterials (ENMs) have shown an effect on morphology, the oxygen carried by erythrocytes, the generation of reactive oxygen species (ROS), coagulation, thrombosis, complement activation, changes in the membrane, and other distressing physicochemical changes, for example, the viscosity of the blood, the longevity of the erythrocytes, and their overall functions [25,26]. Because of the functional characteristics of the erythrocyte membrane cytoskeleton, these cells can be considered good models to study the interactions among nanoparticles and biological membranes. They have average dimensions of 6–8 µm (diameter), with a maximum and minimum thickness of 2.6 µm and 0.8 µm, respectively. Their membranes are formed by glycoproteins and glycolipids (8%), a lipid bilayer (40%), and some embedded globular proteins (52%) that move dynamically in the membrane plane, making it remarkably similar to a 2D viscous fluid. On the extracellular side of the erythrocyte, a ~0.5 mm thick layer (the glycocalyx, formed by glycoproteins and glycolipids) is responsible for cell interactions with its environment. Shape changes can be correlated to the flexibility and deformability of the spectrin cytoskeleton (covalently bound to proteins in the intracellular side of the membrane) when mechanical or rheological forces are applied to the surroundings of the blood cells [27].

### 1.1. Hemocompatibility of Nanoparticles

To evaluate the safety of nanoparticles for biomedical purposes, various hemobiocompatibility tests have been developed. Hemocompatibility is a measure of how a material or device interacts with blood. It evaluates the likelihood of adverse effects from the interaction. A hemocompatible material should not cause adverse reactions such as thrombosis, hemolysis, platelet activation, leukocyte activation, or complement activation [28]. These tests assess different parameters, such as platelet activation, granulocyte response, fibrinolysis, fibrinogen–fibrin conversion, thrombin generation, and complement activation. Together, these parameters provide an overall assessment of the physiology and pathology of the cellular elements of blood, giving a broad but complicated checklist to score [29]. Of these, the only one that specifically evaluates the integrity of red blood cells is hemolysis. Hence, hemolytic activity, which measures the amount of hemolysis caused by different particles or compounds, is one of the most used indicators of hemocompatibility, and it is considered the primary criterion of hemocompatibility. Furthermore, it has been suggested that the mechanism behind hemolysis is membrane damage, as erythrocytes do not have any active uptake mechanism [23].

Hemolysis refers to the loss in the erythrocyte’s membrane integrity, causing the release of intracellular content, mainly hemoglobin, into plasma. Hemoglobin is a crucial protein contained by erythrocytes, and it is the one carrying oxygen to every organ. A loss of hemoglobin can be life-threatening because of the lower amount of oxygen distributed, but their presence outside of the erythrocyte’s membrane can also have detrimental effects. It can be toxic to vascular, myocardial, renal, and central nervous system tissues [30]. The lysis and structural changes in the red blood cell membranes have several pathological effects, such as anemia, kidney function alterations, and jaundice, among others. The hemolytic activity of nanoparticles has been suggested as a marker for hemocompatibility [31]. Varied factors, such as surface charge, nanoparticle morphology, porosity, rugosity, and surface modifications, have an impact on the nanoparticle’s hemocompatibility, either improving it or increasing its hemotoxicity. However, the sole use of hemolysis as a test for hemocompatibility may raise some inconveniences, such as the surge of false positives or false negatives, the sequestering of hemoglobin by the nanoparticles to form a protein corona, the influence of the protein corona on modulating cytotoxicity, and interactions with the red blood cell membranes that modifies their fluid dynamic or flexibility, among several others.

### 1.2. Limitations of Current Tests

The assessment of the hemolytic activity of nanoparticles is the most common test selected to assess the safety of nanomaterials intended for biomedical purposes or for the general evaluation of the nanomaterial’s toxicity, including nanopollutants. It is a rapid and cheap in vitro test that requires only a small amount of blood, and although it is considered highly representative of a real environment, there are some concerns about whether this test can be used to determine the extent of the damage. For example, some nanoparticles can interfere with absorbance lectures or may kidnap hemoglobin on their surface, throwing false positive or false negative results. Another aspect left aside is that some nanoparticles might kidnap hemoglobin on their surface, giving false negative results. A very well-planned methodology to assess hemolytic activity associated with nanoparticles was proposed by Neun and Dobrovolskaya [32]. The initially proposed methodology did not include the incubation of controls and nanoparticles with blood, but a later modification of the methodology included such controls [30]. However, there are other problems that are not being addressed in many of these tests. First, some hemolytic activity methodologies use buffer instead of blood plasma. This means the protein formation and its impact on cytotoxicity, which acts as a fluid barrier, is not considered, resulting in unreliable results [31,33,34]. Second, the anticoagulant selection can affect the protein corona formation and hemolytic activity [35,36]. Third, while many parameters have been contemplated to evaluate nanoparticle hemocompatibility, the morphological evaluation has been left aside. Therefore, there is a considerable risk of missing valuable information on nanoparticle safety or a nanopollutant’s possible toxicity. A fourth shortage of such tests is related to the difficulties arising from the translation of tests designed for biomedical environments into toxicological areas. Unlike nanomaterials designed for biomedical applications that have specific applications and controlled doses, our exposure to nanopollutants is chronic and cumulative, and one concern is how we can assess that effect [9,37]. In addition to the hemolysis test, studying the morphological changes induced by nanopollutants can provide information on potential short- and long-term effects. Erythrocyte morphology is related to the functionality and survival of these cells. It is, therefore, advisable to include morphological assessment, which is quick and inexpensive and can provide an untold part of the story. 

In this work, we follow the hemolysis of whole blood samples exposed to four different concentrations (0.005, 0.01, 0.05, 0.1 mg/mL) of model nanopollutants (silica, magnetite, and carbon nanoparticles), using a modified clinical method, and compared the morphological changes caused by the nanoparticles to the red blood cells at 0 and 24 h after the exposition. A discussion on the potential causes of the observed morphological changes, as well as an interpretation of the analyzed hemolysis behavior, is presented.

## 2. Materials and Methods

Materials: All reagents and solvents, reactive grade, were purchased from Merck-Sigma Aldrich and used as received.

Human blood samples: All human blood samples gathered for this study were from healthy volunteers and used with institutional bioethics approval. Inclusion criteria comprise healthy men or women between 20 and 45 years of age with RhO^+^ blood type. As exclusion criteria, we included the presence of obesity, allergies, pregnancy, hormone imbalance, and smoking habits. For each nanopollutant to test, blood samples were taken from 3 different donors using 6 mL heparinized tubes under sterile conditions; the samples were combined in a 50 mL falcon tube. From this blood pool, a control curve was constructed for each test, ensuring it could be utilized for nanopollutant testing. Using a pool of blood samples reduces the variability in protein corona formation due to individual differences.

### Methods

Synthesis of model nanopollutants: Silica (SiO_2_) nanoparticles were synthesized following a modification of the Stöber method previously reported [38]. Then, 35 mL of ethyl alcohol and 15 mL of deionized (DI) water were mixed with magnetic stirring. To the mixture, 1.5 mL of TEOS was added and magnetically stirred for 1.5 h at 60 °C. Then, 6 mL of NH_4_OH was added dropwise for 5 min, keeping the magnetic stirring and temperature constant. The mixture was kept under magnetic stirring at 60 °C for another 5 h. After this, the solvent was evaporated under continuous stirring (45–50 °C) until dry. After that, the white powder was dispersed in 15 mL of DI water, dried, and finally ground in an agate mortar. Magnetite (Fe_3_O_4_) nanoparticles were prepared, following a previously reported method [39]. Then, 0.44 g (1.63 mmol) of FeCl_3_·6H_2_O was added to 8 mL of DI water and mixed under magnetic stirring for 10 min. In a different reactor, 0.176 g (0.88 mmol) of FeCl_2_·4H_2_O was dissolved in 8 mL of DI water and mixed under magnetic stirring for 10 min. Then, the solution containing the Fe (II) precursor was poured quickly on the Fe (III) solution, and the mixture was heated at 60 °C and stirred for 30 min. After this, 2 mL of NH_4_OH was added dropwise, and stirring was maintained for another 30 min at 60 °C. A black precipitate formed almost immediately. The precipitate was magnetically decanted and washed twice with a mixture of water/ethanol (1:1) and dried in a vacuum oven at 80 °C for 40 min. Finally, carbon nanoparticles (CNPs) were obtained from the deposition of soot from a burning candle flame on soda glass, following a simple procedure reported by Liu and coworkers in 2007 [40]. All three model nanopollutants were fully characterized and were sterilized with UV light before each experiment. 

Characterization: Powered samples of the nanoparticles were characterized by Fourier-transform infrared spectroscopy (FTIR) using a Carey 630 spectrophotometer from Agilent Technologies equipped with a diamond ATR detector. Raman scattering spectra were recorded on a Horiba (Horiba Scientific, Irvine, CA, USA) Raman microscope, XPlora-Plus model, with a green laser of wavelength 523 nm and automated platform; spectra were recorded at 0.01 mW power. Raman samples were prepared by placing samples on a glass substrate. A high-resolution scanning field emission electron microscope (HR-FESEM) (Tescan, MAIA 3, operating at 10 keV, equipped with a Bruker Xflash 6 | 30 Energy Dispersive X-ray detector, EDS) was used for morphological and chemical analyses. Average particle size and size distribution were analyzed using ImageJ software after counting 327 particles (Fe_3_O_4_), 330 particles (SiO_2_), and 368 particles (CNPs), respectively. The dispersive energy X-ray analysis (EDS) was used to determine composition and measure the distribution of elements. Average particle size, size distribution, and zeta potential measurements of 0.1 mg of each sample dispersed in either pure water or PBS solution were obtained using dynamic light scattering equipment (DLS, Microtrac Nanotrac Wave II). No significative changes in particle size distribution were found for measurements performed either in water or PBS.

Erythrocyte osmotic fragility test: For typical hemolysis assessments, a single concentration is tested [30]. However, exposure to airborne nanopollutants in everyday life can vary depending on factors such as proximity to the source, type of weather, and chronicity of the exposure among others [5,16,41,42]. Therefore, we conducted all tests, and analyses at both 0 and 24 h, using the following concentrations: 0.005 mg/mL, 0.01 mg/mL, 0.05 mg/mL, and 0.1 mg/mL. This range was defined to cover a minimal range of concentrations where environmental toxicity has been previously reported for different types of nanoparticles [43,44]. With the intention of having a more accurate overview of the hemolytic activity of model nanopollutants, a clinical protocol for osmotic fragility was modified. Briefly, the normal assay was used as a control, and it was replicated by adding 50 µL of nanoparticle solution (or PBS for the controls) for each concentration assessed at 0 h. For the 24 h test, 900 µL of blood was placed in 2 mL Eppendorf tubes, and an equal volume of nanoparticle solution (or PBS for the controls) was added before incubating at 36 °C. For both 0 h and 24 h tests, the mixture was left to rest for 30 min and then centrifuged at 2500 rpm for 5 min. A total of 100 µL of the supernatant was placed in a 96-well plate according to Figure S1 (see Supplementary Information). 

Hemoglobin adsorption in NPs: For the hemoglobin adsorption assay, 1 mL of PBS was added to an Eppendorf tube and used as a negative control solution. Then, 500 µL of supernatant from the maximum free hemoglobin was taken from the tube of the control sample (corresponding to 100% hemolysis) and placed in an Eppendorf tube along with 500 µL of PBS to create the positive control solution. Moreover, 500 µL of supernatant with 100% free hemoglobin from control samples were added to an Eppendorf tube along with 500 µL of nanoparticles diluted in PBS and repeated for each concentration to test. For each nanoparticle concentration, an Eppendorf tube was filled with 500 µL of nanoparticle solution and 500 µL of PBS to create a nanoparticle interference control. All tubes were placed in the centrifuge and turned on at 2500 rpm for 5 min. Then, 100 µL were taken from each tube and placed in the same microplate (in triplicate). The 96-well plate was taken to the microplate reader, and the absorbance of the supernatants was measured at 550 nm. The same procedure was repeated for the 24 h test. 

Statistical analysis: All assays were performed in triplicate in 96-well transparent round bottom microplates. Statistical differences between groups were determined by one-way analysis of variance (ANOVA) test to compare mean values of hemolysis percent between different nanoparticle exposition concentrations in order to determine whether there is statistical evidence that the associated population means are significantly different. Summary statistics, statistical analyses, and graph curves were performed using GraphPad Prism 8.0.1 (GraphPad Software, Inc., La Joya, CA, USA).

Morphological analysis and EDX: To prevent interference from hypotonic solutions in the morphological analysis of erythrocyte–nanoparticle interactions, 50 µL of blood was directly taken from the pool and mixed in a 1:1 ratio with either PBS or nanoparticles in duplicate Eppendorf tubes. One set was then incubated at 36 °C for 24 h, while the other set was allowed to rest for 30 min before a drop of each concentration was taken and smeared onto a slide. The slides were then stained with Giemsa and analyzed in SEM and EDS without any additional sample preparation.

## 3. Results

Synthesis and characterization of model nanopollutants: Silica (SiO_2_), magnetite (Fe_3_O_4_), and carbon nanoparticles (CNPs) were successfully synthesized as models of nanopollutants commonly found in polluted air at urban locations. The nanoparticles were fully characterized, and their physical characteristics are summarized in Table 1. All nanoparticles are under 50 nm, suitable for having physical interactions with cell membranes or undergoing uptake [19,45,46].

**Figure 2:**
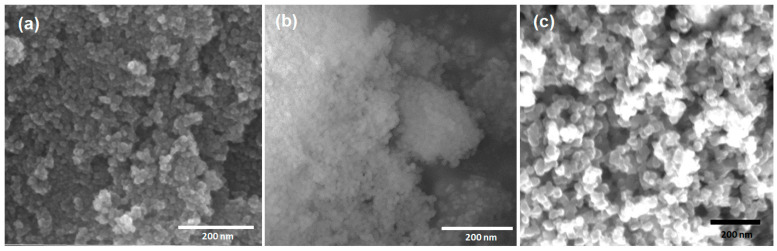
SEM images of the model nanopollutants synthetized. (**a**) Spherical Fe_3_O_4_ nanoparticles (average size ~17.4 nm); (**b**) spherical SiO_2_ nanoparticles (average size ~27.8 nm); (**c**) spherical carbon nanoparticles (CNPs) (average size ~25.4 nm).

FTIR and Raman spectroscopy characterization showed the typical vibrational bands expected for the unmodified materials, as previously reported [38,39,40]. For SiO_2_, the expected Si-O-Si band at 1055 cm^−1^, as well as Si-O-H groups at 950 cm^−1^, were detected by FTIR, while one band at 480 cm^−1^ was detected in the Raman spectra. For Fe_3_O_4_, the FTIR spectra showed the characteristic Fe-O band at 544 cm^−1^, while in Raman, it occurred at 676 cm^−1^. Finally, for CNPs, the FTIR spectra showed some weak bands around 3737, 1550, and 1019 cm^−1^, corresponding to vibrations for O-H, C-C, and C-O groups. In the Raman spectra, the characteristic D and G bands at 1355 and 1600 cm^−1^, respectively, were found, indicating that these particles are graphitized, although the ID/IG ratio (~1.5) reveals the existence of defects on the samples, as expected from the preparation method. Figure 2 presents some selected SEM images for each type of nanomaterial, showing their size, morphology, and general physical characteristics. EDS analysis of the samples indicates the expected chemical composition for each sample and agrees with previous works (SiO_2_: Si, 21.6%; O, 61.2%; Fe_3_O_4_: Fe, 24.2%; O, 41.9%; CNPs: C, 98.1%; O, 1.8%).

**Figure 3:**
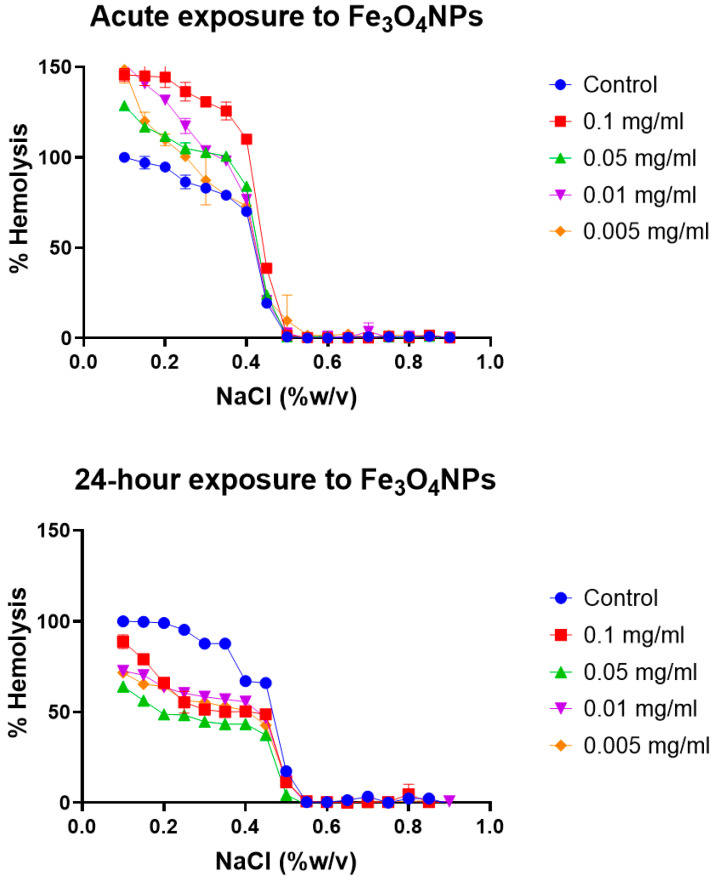
Osmotic fragility curves after acute and 24 h exposure to different concentrations of Fe_3_O_4_ NPs. Different effects are present at time of exposure dependence. At acute exposure, the erythrocytes reach 50% hemolysis sooner than the control but inside the normality range, as shown by the small curve shift towards the right. In contrast, at 24 h exposure, all blood samples reached 50% hemolysis outside of the normality range, showing a curve shift towards the left. Average values from three experimental replicates are presented with error bars (SD) included in plots.

**Figure 4:**
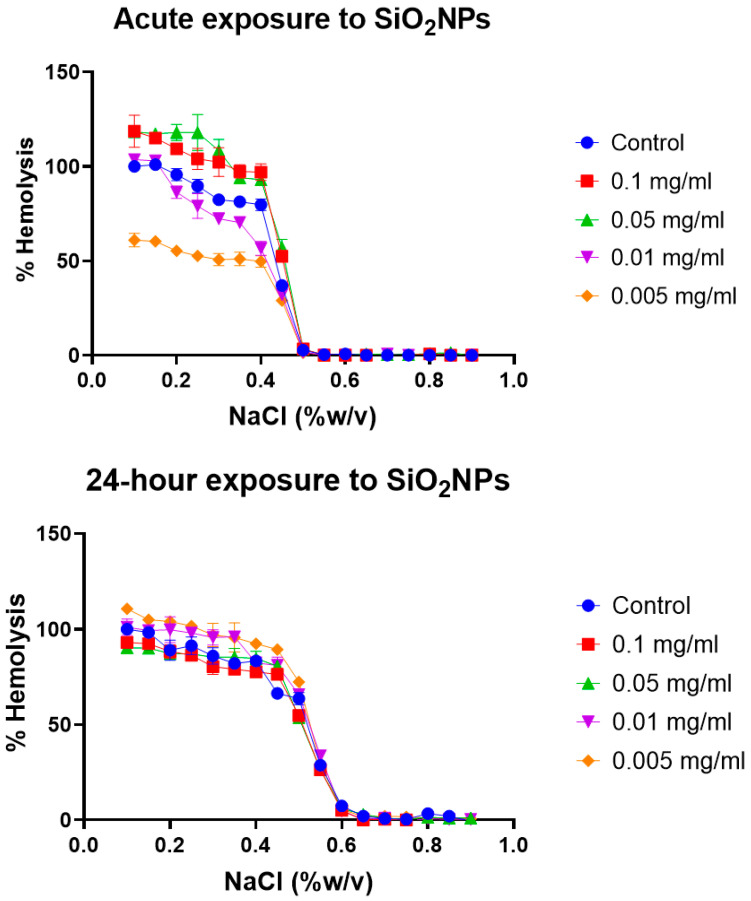
Osmotic fragility curves after acute and 24 h exposure to different concentrations of SiO_2_. Different effects are present at time of exposure dependence. For acute exposure, the lower concentrations do not reach 50% hemolysis in the expected range, they show an increase in their resistance, and 0.005 mg/mL never fully hemolyzes. After 24 h exposure, the osmotic fragility curve normalizes. Average values from three experimental replicates are presented with error bars (SD) included in plots.

**Figure 5:**
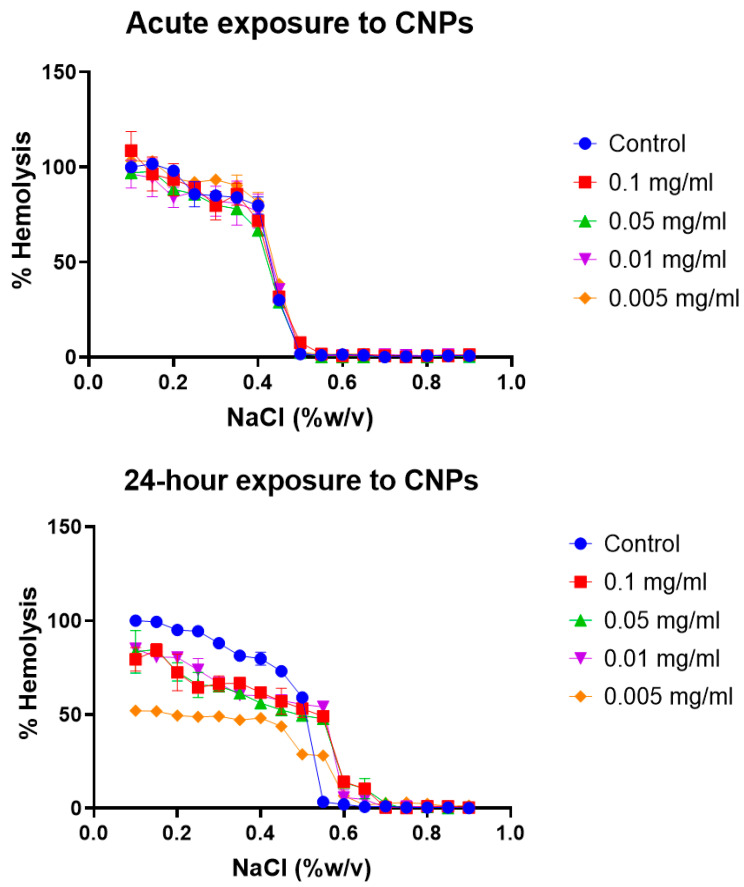
Osmotic fragility curves after acute and 24 h exposure to different concentrations of CNPs. Osmotic fragility curves after acute exposure show no shifts for any of the concentrations tested, but after 24 h exposure, the curve for the three higher concentrations shifts toward the right but never fully hemolyze, and the curve for the lower concentration never reaches 50% hemolysis. Average values from three experimental replicates are presented with error bars (SD) included in plots.

Erythrocyte osmotic fragility test: Broadly, the assay determines when 50% of erythrocytes have suffered lysis in a blood sample under osmotic stress conditions. The erythrocytes are mixed with a NaCl solution of decreasing concentration (from 0.9 to 0.0%). Hemolysis is followed by the spectrophotometric measurement of free hemoglobin (Hb) in solution. The range of clinical normality or the absence of membrane pathology for the fragility test implies that 50% hemolysis occurs between 0.4% and 0.445% NaCl at 0 h and between 0.465% and 0.59% NaCl after 24 h. The plotting of the NaCl concentration versus hemolysis percentage is usually known as the OF curve. Variations in the OF curve to higher or lower concentrations of NaCl with respect to a control sample of healthy erythrocytes are an indication of a pathology or an alteration in the resistance to osmotic stress conditions [47]. The OF curves for the different erythrocyte tests at acute (0 h) and after 24 h of model nanopollutant exposition at four different concentrations (0.005, 0.01, 0.05, 0.1 mg/mL) are shown in Figure 3, Figure 4 and Figure 5.

Hemoglobin adsorption: After the spectrophotometric quantification of free hemoglobin before and after exposure to both Fe_3_O_4_ and SiO_2_ nanoparticles, it was found that there was an almost neglectable percentage of hemoglobin removed from the medium, arguably adsorbed on the surface of both model nanopollutants only after prolonged exposures. Table 2 summarizes the average hemoglobin adsorption at acute (0 h) and after 24 h for the different model nanopollutants. 

### Morphological Analysis in SEM and Energy Dispersive X-ray Spectrophotometry

The morphological effects of the interaction between nanopollutants and erythrocytes were specific to each used type. The elemental composition analysis and mapping were performed to identify whether the nanopollutants were associated with the erythrocyte membrane, present in the extruded content of lysed cells, and to confirm their presence in the different samples. A general overview of the findings can be found in Table 3.

## 4. Discussion

As previously mentioned, nanosilica (SiO_2_), nanostructured iron oxides (Fe_3_O_4_), and carbon nanoparticles (CNPs) were selected as the model nanopollutants for this study, as these three systems represent categories of common nanomaterials found in any urban site, coming either from anthropogenic (construction sites, automotive vehicles, industrial activities) or natural sources. Nanosilica has been detected in urban soils [48] and is also easily formed during abrasive erosive processes of construction materials; Fe_3_O_4_ nanoparticles, among other metal oxides, are produced during mechanical wear and corrosive/oxidative processes of metallic materials (car engines and urban infrastructure) [49,50]. Finally, CNPs are produced during the incomplete combustion of fuels, tires, plastics, or biomass [51]. This selection is intended to allow us to assess the individual contribution of specific nanopollutants to the detrimental effects reported in epidemiological studies [5,42,52,53,54,55,56,57,58]. 

Erythrocyte osmotic fragility test: The methodology utilized for the present study includes an accurate control for the possible presence of blood diseases that might generate false positive or false negative results. Hemolysis caused by different types of nanoparticles (polystyrene, titania, magnetite, gold, silver, silica, zincite, and selenium) has been widely reported in the literature [33,37,59,60,61]. The osmotic fragility (OF) test is commonly used to evaluate the erythrocyte membrane and diagnose hemolytic anemia. Nevertheless, it can also be utilized to assess the hemolysis under controlled osmotic-stress conditions in combination with other factors, such as a nanotoxicological agent. However, modifying a clinical methodology to evaluate the osmotic fragility of erythrocytes has the advantage of showing not only hemolytic activity but also changes in membrane fragility or resistance to hemolysis phenomena. This implies a more detailed and complex analysis of the phenomena associated with membrane alterations that support the shift of osmotic fragility curves. In addition, they allow comparable information on the same phenomenon for acute exposures and over a delayed period. Another advantage is that the same analysis can be run for various concentration ranges to obtain a truly robust picture of the effects not only of the type of nanoparticle but also of its concentration and type of exposure.

Magnetite nanoparticles (Fe_3_O_4_): At 0 h, the results of the erythrocytic fragility assay for exposure to four different concentrations of magnetite nanoparticles (0.005, 0.01, 0.05, 0.1 mg/mL) were within the normal range for all concentrations tested. However, after 24 h, 50% hemolysis was not reached within the expected (healthy) range of NaCl concentrations for any of the tested concentrations of Fe_3_O_4_, as indicated by a leftward shift in the curve (Figure 3). Clinically, the shift of the curve to the left occurs in thalassemia when the erythrocytes have a pathological osmotic resistance due to membrane damage including abnormally clustered protein band 3. The membrane damage has been previously associated with oxidative stress induced by free iron [47,62]. However, in this case, the control curve is normal, indicating the absence of pathology. Therefore, any changes observed are related to the interaction or presence of the nanoparticles.

Silica nanoparticles (SiO_2_): After exposure to four different concentrations of silica nanoparticles (0.005, 0.01, 0.05, 0.1 mg/mL), two concentration-dependent behaviors are observed (Figure 4). For nanoparticles with a lower concentration, the osmotic fragility curve shifts slightly to the left, while those with a higher concentration show a shift to the right. Higher concentrations lead to 50% hemolysis occurring at NaCl concentrations outside the normal range, indicating an increase in the fragility of erythrocytes. On the other hand, lower concentrations result in 50% hemolysis occurring within the clinically normal range of NaCl concentrations. This shift to the right is clinically associated with diseases such as spherocytosis, where the erythrocyte membrane shows damage to the lipid bilayer, membrane proteins, and cytoskeletal network. These alterations disrupt the cohesion of the cytoskeleton and lipid bilayer, leading to a loss in surface area and stability [63]. In particular, deficiencies have been observed in spectrin, as well as other proteins, such as ankyrin, nothingness 3, and protein 4.2. These protein deficiencies, along with decreases in phospholipids, cholesterol, and general cell metabolism, negatively impact the elasticity and deformability of the membrane, making it more fragile [63]. After 24 h of exposure to silica, we observed that almost all concentrations showed a rightward shift compared to the control, and the effect observed in the acute-exposure samples is no longer appreciated. This could be attributed to the internalization of particles and the formation of aggregates, as well as changes in the formation of the biocorona. The biocorona may act as a fluid barrier, ultimately protecting the erythrocytes.

Soot carbon nanoparticles (CNPs): According to the osmotic fragility test, there is no change observed in the erythrocytes after acute exposure to CNPs (Figure 5). This could be due to particle aggregation phenomena, which could cause the particle’s sedimentation and further lack of interaction with the cells, or it could even be due to the formation of a protein corona acting as a fluid barrier that diminishes the cytotoxic effects [64,65]. However, after 24 h, the three higher concentrations of soot induce hemolysis sooner, with 50% of the cells experiencing hemolysis. The lowest concentration, on the other hand, does not reach 50% hemolysis at all. In accordance with our results, previous studies have reported that carbonaceous ultrafine particles have minimal hemolytic effects, indicating limited damage to the erythrocyte membrane [60].

Likewise, this test allows the assessment of hemoglobin adsorption on the nanopollutant surface to ensure that such adsorption is not interfering with precise positive or negative results. Furthermore, since the methodology is intended to assess erythrocyte membrane behavior under stress conditions, it allows a fine analysis of nanopollutant interactions with the red cells beyond the black-and-white results of hemolysis. The shifts in the osmotic fragility curves can be associated with different pathological mechanisms well known for the erythrocyte’s membrane integrity with acknowledged diseases and health effects as outcomes. Therefore, having this picture helps to broaden the understanding of the possible mechanisms behind the rise in specific diseases in association with airborne nanopollutants. Also, a single pool of blood can be utilized to evaluate the hemolytic activity and the possible morphological changes induced. This leads to a broader understanding of the interactions between nanopollutants and erythrocytes and a richer interpretation of the hemolytic activity results, specifically for nanoparticles.

The observed alterations may occur when osmotic imbalances happen. However, the most recent hypotheses indicate that the deformations are due to changes in the lipid composition of the membrane. Additionally, changes are observed in the proteins associated with the cytoskeleton that maintain the integrity and flexibility of the membrane in optimal conditions and prevent the occurrence of vesiculation [66].

Hemoglobin adsorption: After the spectrophotometric quantification of free hemoglobin before and after exposure to both Fe_3_O_4_ and SiO_2_ nanoparticles, it was found that there was an almost neglectable percentage of hemoglobin removed from the medium, arguably adsorbed on the surface of both model nanopollutants only after prolonged exposures. Table 2 summarizes the average hemoglobin adsorption at acute (0 h) and after 24 h for the different model nanopollutants. However, for CNPs (which are highly porous materials and, therefore, have a higher surface area rate), the percentage of adsorption is higher as concentration increases. So, to some extent, the variability in the hemolytic activity and osmotic fragility results may be attributed to the adsorption of hemoglobin on the surface of the nanoparticles, which increases with longer exposure times, as shown in Table 2. On this basis, the modified osmotic fragility test in combination with the hemoglobin adsorption control is particularly interesting as it reveals a long-term interaction that can be influenced by the formation of a protein corona. While these variations may not be significant enough to produce false negatives or positives, they suggest that nanomaterials with different levels of surface porosity may have varying impacts that may go unnoticed. This could lead to misleading results in the safety or cytotoxicity and may have clinical relevance when considering concentration and nanoparticle porosity.

The changes observed after 24 h of nanopollutant exposure may also be due to the differences in the proteins adsorbed on the surface of the nanoparticles. Given that the formation of the biocorona is dynamic and may vary depending on the interaction time and differences in the concentration of proteins present in plasma, these small changes may be key to affecting the specific nanopollutant–erythrocyte interaction, including hemolytic activity [9,65,67]. A detailed analysis of the biocorona is outside the objectives of this work but is definitively important to be considered for subsequent research in order to expand our understanding of the processes occurring.

An important limitation is the missing flow-induced mechanical stress naturally occurring in the bloodstream. The mechanical stress of a continuous flow could be sufficient to lyse erythrocytes that had become more fragile after their interaction with the nanopollutants and to assess changes in deformability and differences in nanopollutants uptake due to the movement dynamics [31,68]. This is also a future implementation being contemplated to broaden the data gathered. 

### Morphological Analysis in SEM and Energy Dispersive X-ray Spectrophotometry

**Figure 6:**
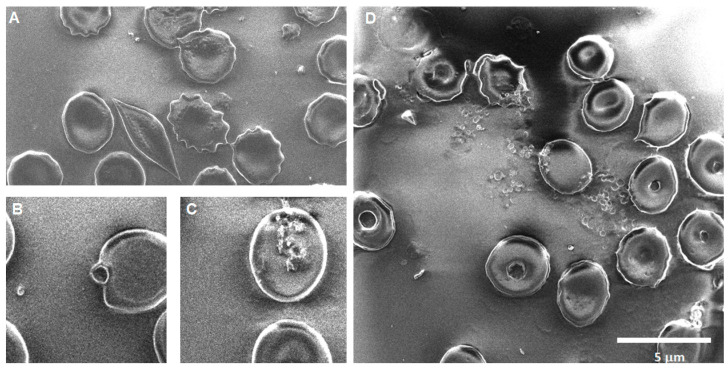
Erythrocytes after acute exposure (0 h) to Fe_3_O_4_. (**A**) A sickle cell can be observed, along with more crenated cells and rough membrane cells. Additionally, small vesicles ~455 nm are present in various areas of the image. (**B**) On the left side, a small vesicle is visible. On the right side, a large bud is formed on the erythrocyte membrane. (**C**) Fe_3_O_4_ NPs are seen to be associated with the surface of erythrocyte membranes. (**D**) Multiple erythrocytes with vesiculation are present, with residues of vesicles surrounding them. Various crenated erythrocytes of different shapes are also visible, with those in the upper left already lysed. Target cells are also present.

**Figure 7:**
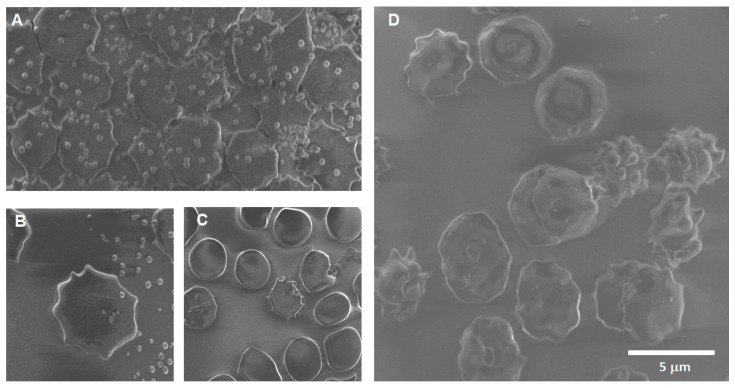
Erythrocytes after 24 h of exposure to Fe_3_O_4_ NPs. (**A**) Crenated erythrocytes can be observed with various deformations of the membrane, which is covered with a large number of extracellular vesicles of ~455 nm. (**B**) Extracellular vesicles of different sizes are observed on the surface of a crenate erythrocyte. (**C**) An erythrocyte bursts, spilling its cellular content to the right side. On the left, a crenate erythrocyte with an aggregate of particles on its surface can be observed, while on the far left, another erythrocyte has aggregates of particles associated with its membrane. (**D**) Multiple erythrocytes with deformations of their membrane, including acanthocytes, erythrocytes, and target cells, can be observed.

Morphological changes after Fe_3_O_4_ nanoparticle exposure: For acute exposure, the main finding besides crenation is the presence of extracellular vesicles of large size at acute exposure (Figure 6B) and a smaller size after longer exposition times (Figure 7A,B). The sheading of vesicles is an event triggered by an intracellular calcium increase due to factors like apoptosis, oxidative stress, senescence, injury, etc. As intracellular calcium levels rise, the floppase, scramblase, and calpain also increase, while flippase, ATP, and antioxidant defenses decrease, allowing the formation of microvesicles and exosomes [69]. The calcium rise also results in a loss in membrane asymmetry because of changes or damage to the lipids, the cytoskeleton detachment from the membrane, and the protein oxidation and clustering, resulting in the aggregation of membrane and membrane-associated proteins like spectrin, ankyrin, and band 3, among others [70]. The formation of extracellular vesicles and the presence of crenation could explain the mechanism behind the observed changes in the fragility curve. The findings can be related to the loss in phospholipid asymmetry and protein anchorage to the cytoskeleton resulting in budding, EVs release, and the erythrocyte membrane general deformation. The sheading of vesicles can also reflect different populations present in the samples; it has been reported previously that vesiculation increases as erythrocytes grow “older”, but the younger erythrocytes are more capable of squeezing out denatured hemoglobin in vesicles [71]. So, the content of the vesicle itself may vary depending on the mechanism driving it and the erythrocyte life stage.

Morphological changes after SiO_2_ nanoparticles exposure: Previous studies assessing silica nanoparticles’ broad blood compatibility, showed severe hemolysis in response to different coating and concentrations (10 μg/mL, 20 μg/mL, and 40 μg/mL) [46]. In contrast, we found a dose-dependent effect in which 5 and 10 μg/mL concentrations did not induce 50% of hemolysis sooner than expected for a healthy control, but 50 and 100 μg/mL only did for acute exposure. Effects can vary also due to the nanoparticle size, surface coating, and the experimental methodology itself. However, this type of result heightens the importance of the measurements at different times and the concentration range selection. Opposite effects can be missed if the range selected is too narrow, and changes in such effects in time are out of the picture if only acute exposures are considered. Valuable information can be derived from the observations. The absence of osmotic fragility after prolonged exposure to silica can imply mechanisms of aggregation, internalization, or protein corona formation. 

**Figure 8:**
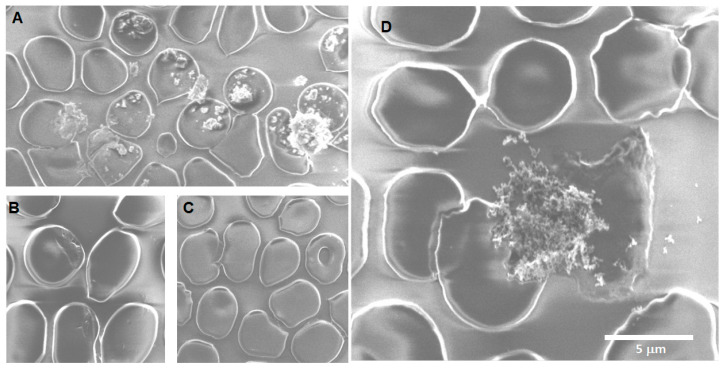
Erythrocytes after acute exposure to SiO_2_ NPs. (**A**) The agglomeration of SiO_2_NPs is visible in association with the cell’s membrane. Some cells are bursting on the right and at the bottom left, while a dacrocyte is present in the center. A small cell at the bottom center may be membrane remains or a schistocyte. (**B**) The presence of notches in the membrane can indicate changes in lipidic conformation. (**C**) An echinocyte is present on the far left. (**D**) A pair of bursting cells can be observed, with the cellular content (including SiO_2_NPs) located between them.

Silica 0 h: Recurring observations in the images obtained for the SiO_2_ samples show areas of particle aggregation in association with cells, bursting cells, crenation, cells with membrane notches, stomatocytes, dacrocytes, and schistocytes (see Figure 8).

**Figure 9:**
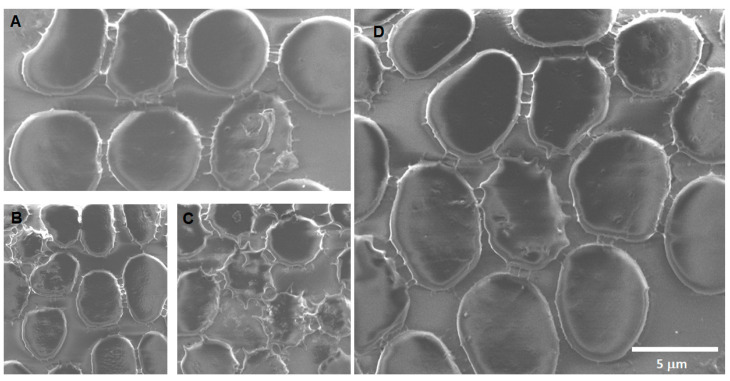
Erythrocytes after 24 h of exposure to SiO_2_ NPs. (**A**) Cells that appear connected through short projections of their membranes, the bright dot in the lower right corner are particles on the surface of the cell membrane. (**B**) An acanthocyte is present in the upper left, all the cells in the image display membranes with ridges and notches on the surface, as well as projections along the edges. (**C**) SiO_2_ NP aggregates cover the echinocytes and acanthocytes. (**D**) The cell in the upper right corner has projections that end in rounded bright tips indicating budding. Some small vesicles of ~70 nm are also present.

Silica 24 h: The main findings after 24 h of exposure of erythrocytes to SiO_2_ nanoparticles are the formation of echinocytes, acanthocytes, agglomerations of SiO_2_ associated with the erythrocyte’s membrane, smaller membrane notches rounded in shape, and membrane projections between cells that seem to be associated with particles at the tip of some projections, as shown in Figure 9.

**Figure 10:**
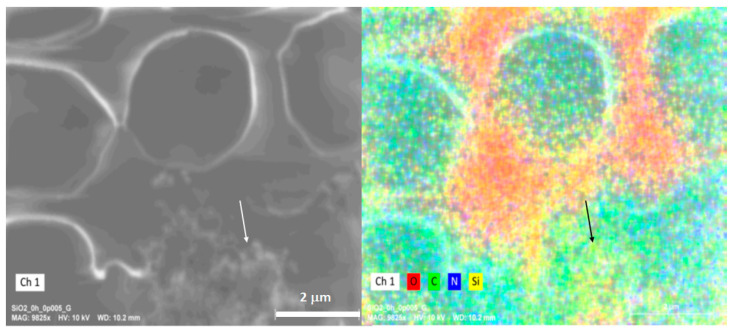
Elemental mapping of erythrocytes exposed to SiO_2_ NPs. The white arrow at the left image indicates the place where some cellular content of a hemolyzed erythrocyte is located. The right image shows an EDS (energy dispersive spectroscopy) elemental mapping, presenting the 2D distribution of oxygen, carbon, nitrogen, and silicon in the selected area, and a black arrow points at the same previously indicated location to trace the amount of Si in it, suggesting that it contains SiO_2_ nanoparticles.

The elemental mapping (Figure 10) shows the presence of elements associated with cellular content and Si, as shown in the following figure. The presence of higher concentrations of Si in the cellular content after a short exposure is evidence of the passive internalization of the SiO_2_ NPs by the erythrocytes. However, no information on the mechanism was gathered.

Morphological changes after CNP exposure: Our morphological analysis provides further insights into the full picture; the presence of extracellular vesicles and the alteration in erythrocyte shape suggest subtle damage to the membrane. According to the biological mechanisms behind the morphological findings [69,72], oxidative stress, changes in phospholipid structure, and loss in protein anchorage to the cytoskeleton are the possible underlying mechanisms triggered by CNPs; both lipid peroxidation and oxidative injury have been observed in erythrocytes exposed to carbonaceous nanoparticles [60] (Pan et al., 2020). Another finding is the budding of echinocytes releasing vesicles under 100 nm in size. The presence of extracellular vesicles is accompanied by the appearance of activated platelets. The platelets are considered part of the immune system since they have been reported to produce soluble factors that interact with immune cells, leading to inflammation, and when pathologically activated, they can promote tissue damage and fibrosis [73]. 

**Figure 11:**
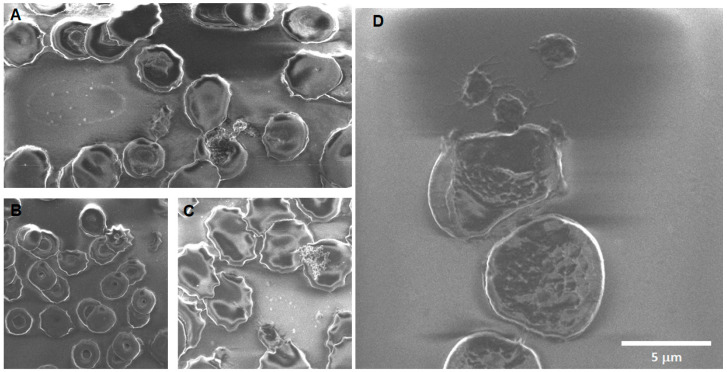
Erythrocytes after acute exposure to CNPs. (**A**) In the lower right of the image, a bursting erythrocyte is shown with its cellular content exposed. Most of the cells show crenation, and there is also a piece of broken membrane in the center. The probable remains of a leukocyte cell in the left area of the image. (**B**) The image also shows a large number of stomatocytes, with an acanthocyte located at the upper right next to a broken membrane. (**C**) There is a significant amount of nanovesicles ~70 nm on top of the membrane as well as activated platelets near soot aggregates. (**D**) The membranes of the cells appear rough and have vesicle-shaped indentations. One of the cells is lysed and appears to be in close proximity to activated platelets.

Soot 0 h: The images of acute exposure to CNPs (Figure 11) show a great extent of damage to the membrane, among which we can mention crenation, rupture, the appearance of roughness on the surface, the formation of stomatocytes, and even some round notches observed on the surface of the membrane. Furthermore, an unexpected discovery struck, the presence of activated platelets.

**Figure 12:**
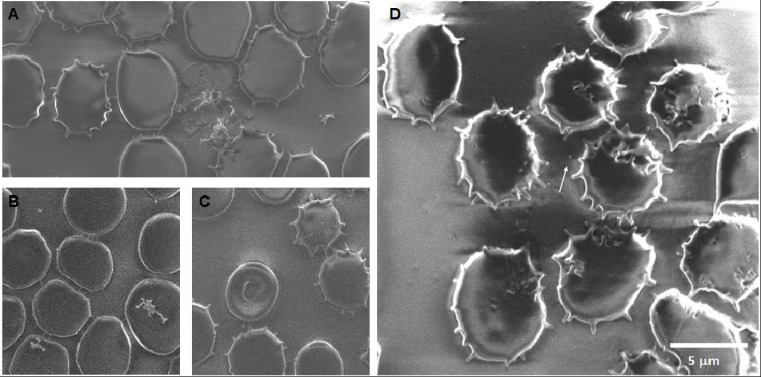
Erythrocytes after 24 h of exposure to CNPs. (**A**) A bursting cell can be seen in the lower right part of the image, with its cellular contents spilling to the left and to the right, and an aggregate of CNPs is observed. There are multiple cells with membrane projections at what appears to be the beginning of crenation. (**B**) Budding that creates adherences between cells and large number of vesicles ~70 nm on top of the membrane of erythrocytes at the bottom right and left. (**C**) A target cell is observed in the central part and echinocytes around it. (**D**) There are multiple cells with projections, and the white arrow points to what appears to be a detached tip of the projections.

Soot 24 h: The main findings after 24 h of CNP exposition (Figure 12) are evidence of carbon nanoparticle internalization, extensive crenation, formation of membrane projections that create adherences between cells, formation of extracellular vesicles of around 70 nm on top of the erythrocyte’s membranes, and formation of echinocytes.

**Figure 13:**
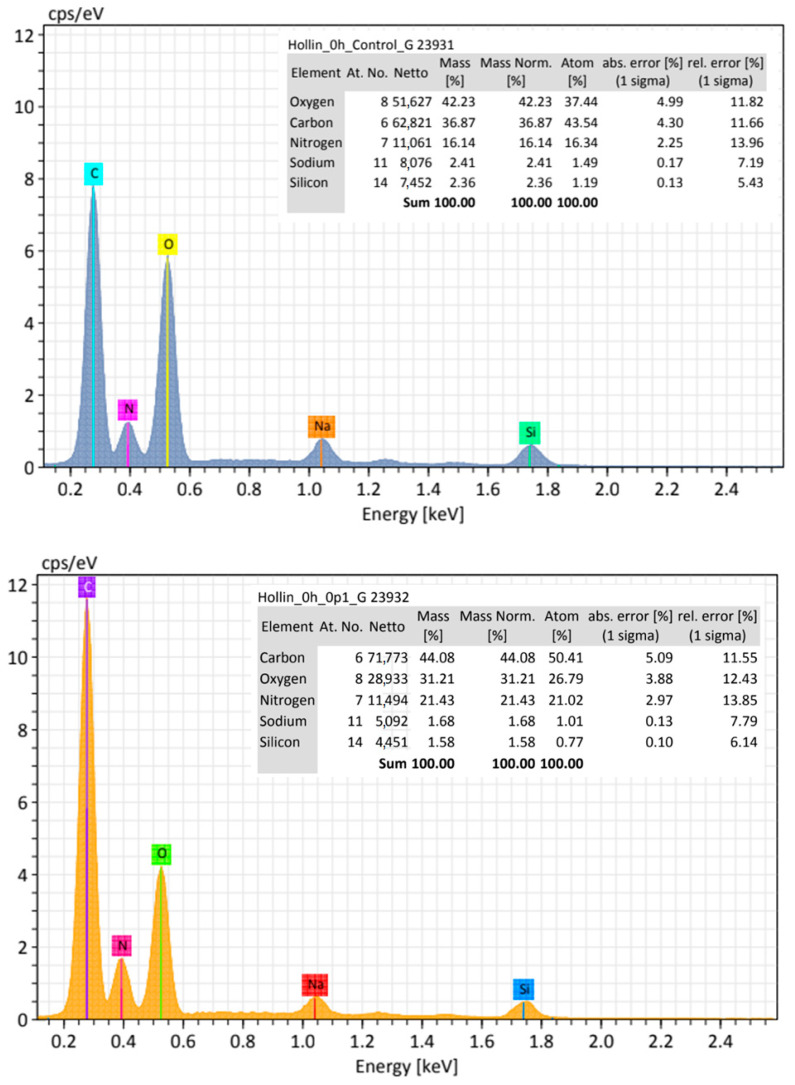
EDX analysis. The left image shows the EDX results for an erythrocyte in the control group, and the right image the results for an erythrocyte exposed to 0.1 mg/mL of CNPs.

As shown in Figure 13, the compared elemental analysis between erythrocytes from the control and erythrocytes exposed to 0.1 mg/mL of CNPs shows that the carbon, oxygen, and nitrogen proportions change drastically, with carbon content increasing for CNPs exposed erythrocytes, which suggest the presence of CNPs on the surface of the membrane even when there is no conspicuous aggregate on top of the erythrocyte analyzed.

From the morphological findings, the sheading of extracellular vesicles is a key observation. It has been reported that erythrocyte extracellular vesicles can alter neutrophil’s phagocytic behavior depending on their size and content [74]. Extracellular nanovesicles released by human erythrocytes have been considered to play different roles in physiological, pathological, and even immune processes; their formation has been linked to intracellular calcium level increases, ATP depletion, or oxidative stress conditions [69]. They also have been reported to occur after several days of storage or gamma-radiation exposure, indicating membrane structure alterations that may affect functionality, leading to hemolysis [75]. This means that erythrocytes play an active role in homeostasis and inflammation through extracellular communication relaying on vesicle content. Furthermore, a rise in the number of vesicles released has been associated with diverse cardiovascular and immune diseases including hypertension, thrombocytopenia, multiple sclerosis, sickle cell anemia, acute myocardial infarction, acute ischemic stroke, hyperlipidemia, metabolic syndrome, diabetes, and atherosclerosis [69,76,77]. To mark their contribution to homeostasis, Tissot and colleagues mention that erythrocyte’s smaller vesicles can remove dangerous molecules from the erythrocyte allowing their normal survival [78]. Therefore, the formation of the erythrocyte’s vesicles after exposure to nanopollutants may respond to a homeostatic mechanism, both clearing the internalized particles and sending messages to the surrounding immune cells. The implication for human health from vesicle increases can shed light on the mechanisms behind the rise in specific diseases in association with airborne nanopollutants.

## 5. Conclusions and Perspectives

Osmotic fragility studies show that exposure to different types of nanopollutants has different concentration-dependent effects. Morphological analyses allow us to identify that there are changes in the integrity of the membrane associated with different phenomena linked to inflammatory processes mediated by the immune response and various vascular pathologies. Therefore, this work shows that the absence of hemolysis is not always an indicator of the absence of damage. It must be considered that hormesis phenomena may occur, so including a scan of various concentrations in hemocompatibility tests is essential. In addition, morphological analyses that require little time and are not expensive can be implemented to have robust information on the toxicological profiles of both nanopollutants and nanomaterials used for biomedical applications. The findings help to strengthen the evidence of the mechanisms behind the relationship between nanopollutants and the increase in cardiovascular diseases. 

Although osmotic fragility is a time- and manual-consuming technique, it remains used for human blood evaluation because of the clinical application and low cost. In our study, we used this tool with some modifications to evaluate the erythrocyte vulnerability in an airborne nanopollutant model. While the results are interesting, there are some limitations of this study, for example, the biochemical mechanisms and other membrane effects were not evaluated. Eventually, we must analyze whether reactive oxygen species along with reactive nitrogen species are related to the osmotic imbalance and how cytoskeleton proteins and membrane molecules are affected. Additionally, it is necessary to evaluate how membrane shape, size, and roughness changes are associated with osmotic instability and the acute and chronic potential exposition. In further studies, it could be interesting to evaluate other erythrocyte indices and detect early deviations in size, volume, or shape to help us understand the possible mechanisms involved.

This work is motivated by the need to design better and specific protocols to evaluate the effects of nanopollutants in acute, subchronic, and chronic conditions and the implications they may generate, triggering different pathologies, such as immune, cardiovascular, or neurodegenerative diseases. Therefore, although the experimental methodology here used may have several limitations, as it was designed for the clinical evaluation of hemolysis to determine the existence of a blood pathology, it was not designed for the evaluation of blood cell functionality impacts by environmental nanopollutants. However, this work indicates that an adapted methodology may help to easily identify potential risks associated with human exposition to environmental nanostructured contaminants. Current methods only consider hemolytic activity at a unique concentration and time, without including simultaneous cell morphology analyses. Recent evidence supports a more active role of erythrocytes in immune response, and the morphological evidence found supports this role as a potential messenger through vesicle releasing, going beyond its traditional oxygen transport function. Erythrocytes may take part in the activation of different pathologies after the loss of homeostasis when interacting with nanoparticles. Further research has to be performed in order to better understand this impact and its physiological consequences.

## Figures and Tables

**Table 1:** Main characteristics of synthetized model nanoparticles.

Nanoparticle	Diameter (DLS)	Polydispersion Index (PDI)	Size (SEM)	Morphology	Zeta Potential
Magnetite	60.7 nm	0.256	10 nm	Spheric	+21.1 mV
Silica	32.4 nm	0.559	26 nm	Spheric	−80.9 mV
Candle Soot	71.1 nm	0.115	25 nm	Spheric	−32.2 mV

**Table 2:** Indirect measure of hemoglobin adsorption on nanopollutant’s surface. Normalization was performed on data averaged from three experimental replicates using PBS as positive control. The percentage (%) of hemoglobin is the amount of hemoglobin detected in a supernatant; any diminish in the percentage after interaction with nanoparticles at different concentrations is assumed to be adsorbed in the surface of the particles.

Average Acute Exposure Adsorption						
**Nanopollutant**	Positive Control	0.1 mg/mL	0.05 mg/mL	0.01 mg/mL	0.005 mg/mL	Adsorption %
Magnetite	100	98.75 ± 3.5 *p* = 0.918	98.74 ± 1.1 *p* = 0.405	98.24 ± 2.4 *p* = 0.625	98.48 ± 3.4 *p* = 0.858	1.25–1.76%
Silica	100	99.38% ± 2.4 *p* = 0.967	100 ± 1.1 *p* = 0.743	99.34 ± 2.4 *p* = 0.960	100 ± 3.6 *p* = 0.705	0.62–0.66%
CNPs	100	78.10 ± 22.2 *p* = 0.751	90.09 ± 7.8 *p* = 0.637	87.46 ± 13.9 *p* = 0.791	99.23 ± 5.8 *p* = 0.999	0.77–21.9%
**Average 24-h Exposure Adsorption**						
**Nanopollutant**	**Positive Control**	**0.1 mg/mL**	**0.05 mg/mL**	**0.01 mg/mL**	**0.005 mg/mL**	**Adsorption %**
Magnetite	100	100 ± 1.0 *p* = 0.446	100 ± 1.0 *p* = 0.446	98.91 ± 1.0 *p* = 0.446	100 ± 3.0 *p* = 0.609	1.09%
Silica	100	100 ± 1.0 *p* = 0.978	100 ± 1.9 *p* = 0.997	99.78 ± 3.2 *p* = 0.999	98.63 ± 4.2 *p* = 0.933	0.22–1.63%
CNPs	100	86.04 ± 12.8 *p* = 0.406	90.75 ± 31.2 *p* = 0.948	97.38 ± 7.9 *p* = 0.931	95.06 ± 32.2 *p* = 0.994	2.65–13.96%

**Table 3:** Findings of morphological analysis after erythrocytes exposure to model nanopollutants *.

System/ Effect	Fe_3_O_4_		SiO_2_		CNPs	
	0 h	24 h	0 h	24 h	0 h	24 h
Crenation (%)	10–30	11–45	13–50	51–70	16–50	44–92
Extracellular vesicles (EVs)	+ ^a^	+ ^b^	−	+ ^c^	+ ^d^	+ ^e^
Rough membranes	−	−	+	+	+	+
Lysis	+	+ +	+	+ +	+	+
Particles on membrane	+	+	+	+	+	+
Notches on membrane (%)	<1	<1	<1	<1	11–14	<1
Cell-adhesion	−	+	−	+ + +	+	+ +
Echinocytes	−	−	+	+ +	−	+ + +
Activated platelets	+	+ +	−	−	+ +	+ + +

* Control did not show any of the morphological features found for blood exposed to model nanopollutants. (−) indicates no observed changes respect control; (+), (+ +), or (+ + +) indicates the observation of a low, moderate, or large number of events related to that event, with respect to control. ^a^ Large number of 800–1000 nm EVs; ^b^ Great number of ~455 nm EVs; ^c^ Small number of ~70 nm EVs; ^d^ Small number of 70–455 nm EVs; ^e^ Large number of 70 nm EVs.

## Data Availability

Data are contained within the article and supplementary materials. Other data presented in this study are available on request from the corresponding author (M.A.M.-R.).

## Supplementary Materials

The following supporting information can be downloaded at: https://www.mdpi.com/article/10.3390/toxics12010092/s1, Figure S1: Sample distribution of the 96-well plate spectrophotometric measurements for the erythrocyte osmotic fragility test.
