# Supplementary material for: Erythrocyte Vulnerability to Airborne Nanopollutants
Source: Toxics. 2024 Jan 21;12(1):92. doi: 10.3390/toxics12010092 (PMC10818893; doi:10.3390/toxics12010092)
Supplement: Supplementary file 1 [file toxics-12-00092-s001.zip › toxics-2802400-supplementary.pdf]

# Supplementary Materials: Erythrocyte Vulnerability to Airborne Nanopollutants

*Erythrocyte osmotic fragility test:* Sample distribution for the 96-well plate spectrophotometric measurements for erythrocyte osmotic fragility test.

| | 1 | 2 | 3 | 4 | 5 | 6 | 7 | 8 | 9 | 10 | 11 | 12 |
|---|---|---|---|---|---|---|---|---|---|---|---|---|
| A | NP 0.1 | NP 0.1 | NP 0.1 | NP 0.05 | NP 0.05 | NP 0.05 | NP 0.01 | NP 0.01 | NP 0.01 | NP 0.005 | NP 0.005 | NP 0.005 |
| B | T1 | T1 | T1 | T2 | T2 | T2 | T3 | T3 | T3 | T4 | T4 | T4 |
| C | T5 | T5 | T5 | T6 | T6 | T6 | T7 | T7 | T7 | T8 | T8 | T8 |
| D | T9 | T9 | T9 | T10 | T10 | T10 | T11 | T11 | T11 | T12 | T12 | T12 |
| E | T13 | T13 | T13 | T14 | T14 | T14 | T15 | T15 | T15 | T16 | T16 | T16 |
| F | T17 | T17 | T17 | | | | | | | | | |
| G | H + 0.1 | H + 0.1 | H + 0.1 | H + 0.05 | H + 0.05 | H + 0.05 | H + 0.01 | H + 0.01 | H + 0.01 | H + 0.005 | H + 0.005 | H + 0.005 |
| H | H | H | H | | | | | | | PBS | PBS | PBS |

**Figure S1.** 96 well plate display. T1 is the tube with 0.1% NaCl, T2 has a 0.15% NaCl, T3 has a 0.2% NaCl, T4 has 0.25% NaCl, T5 has 0.3% NaCl, T6 has a 0.35% NaCl, T7 has a 0.4% NaCl, T8 has a 0.45% NaCl, T9 has a 0.5% NaCl, T10 has a 0.55% NaCl, T11 has a 0.6% NaCl, T12 has a 0.65% NaCl, T13 has a 0.7% NaCl, T14 has a 0.75% NaCl, T15 has a 0.8% NaCl, T16 has a 0.85% NaCl, T17 has a 0.9% NaCl, H corresponds to the 100% free hemoglobin supernatant or positive control for the adsorption assay, PBS corresponds to the 0% free hemoglobin or negative control for the adsorption assay, H + 0.1 to H + 0.005 are the 100% hemoglobin supernatant mixed with the nanopollutant solutions at the different concentrations tested, NP 0.1 to NP 0.005 are the nanopollutants solutions diluted in PBS at the different concentrations tested to asses possible nanoparticle interference.
